# Tactile Image Sensors Employing Camera: A Review

**DOI:** 10.3390/s19183933

**Published:** 2019-09-12

**Authors:** Kazuhiro Shimonomura

**Affiliations:** Department of Robotics, Ritsumeikan University, 1-1-1 Noji-Higashi, Kusatsu, Shiga 525-8577, Japan; skazu@fc.ritsumei.ac.jp; Tel.: +81-77-561-4826

**Keywords:** tactile sensor, camera, image sensor, tactile perception, contact detection, force sensing

## Abstract

A tactile image sensor employing a camera is capable of obtaining rich tactile information through image sequences with high spatial resolution. There have been many studies on the tactile image sensors from more than 30 years ago, and, recently, they have been applied in the field of robotics. Tactile image sensors can be classified into three typical categories according to the method of conversion from physical contact to light signals: Light conductive plate-based, marker displacement- based, and reflective membrane-based sensors. Other important elements of the sensor, such as the optical system, image sensor, and post-image analysis algorithm, have been developed. In this work, the literature is surveyed, and an overview of tactile image sensors employing a camera is provided with a focus on the sensing principle, typical design, and variation in the sensor configuration.

## 1. Introduction

Tactile sensing in fingers and hands has an important role in the identification and manipulation of objects for humans and robots [1]. For robotic tactile sensing, various types of transduction methods have been developed. For example, a resistive-based sensing device (e.g., a strain gauge), capacitive-based sensor (e.g., a conductive polymer), optical-based sensor, magnetism-based sensor, and piezoelectric-based sensor are commonly used [2,3]. Some sensors are commercially available and have been used for robotic manipulation [4,5]. Most of these typical tactile sensors provide a force at the single point or averaged force in the measurement area, and the spatial resolution they provided can be low, even if multiple sensors are arranged together. Therefore, it may be difficult to measure a detailed distribution about the contact and to know the position and orientation of the object from the sensor outputs.

Tactile sensing in humans is basically distributed or arrayed to obtain not only the averaged force applied but also the position and shape of the contact area. According to [1,6], tactile sensors with similar performance to a human fingertip should have 1 to 2 mm of spatial resolution and 50 to 100 sensing points. Spatial resolution and the number of tactile sensing elements (sometimes referred to as “taxels”) can be important factors for sophisticated robotic tactile sensing. In fact, the effectiveness of arrayed tactile sensors and the importance of spatial resolution of robotic tactile sensors have been reported [7,8].

Arrayed tactile sensors, such as polymer-based arrayed tactile sensors [9], pressure conductive rubber-based sensors [10,11], and piezoresistive rubber-based sensors [12] have also been studied. Furthermore, tactile sensor modules that can be arranged on the target surface [13,14], and arrays of mechanical tactile elements with elastic elements [15] have been proposed. The number of tactile elements in these sensors is limited to up to several tens. Silicon microelectromechanical system (MEMS)-based arrayed tactile sensors [16] and piezoelectric polymer microelectrode array (MEA)-based sensors [17] can realize a smaller pitch and larger number of tactile elements. When the number of tactile elements increases, wiring for readout from a large number of sensing elements generally becomes a problem for these sensors. Parallel wiring is not suitable for connecting the sensor to a post-processing system; however, scanning methods can be used. For this, a scanner circuit should be prepared and integrated with the sensing block. Usage of video signal output to read out the output from resistive-based tactile sensor arrays has been proposed [18]. The point that tactile information is read out as an image is the same idea as that of a tactile image sensor using a camera, which is the focus of the present work.

Can cameras be used more directly to obtain tactile information? If it is possible to use a camera, which consists of an optical system and image sensor, for tactile sensing, finer pixel pitch and a large number of sensing points can be easily realized. Moreover, a readout scanner is commonly integrated in the image sensor; it can easily read out the response, even from millions of pixels. This idea was proposed more than 30 years ago [19,20,21], and there have been many studies on tactile sensing using a camera since then. Recently, cameras have become more sophisticated and less expensive, and, also, the use of a camera has become more convenient, because a general-purpose interface, such as USB, can be used for the connection. Additionally, the evolution of image processing hardware and software makes it easier to use image processing, even in real-time and embedded applications. With this background, recently, research on tactile sensors using cameras has increased. Its applications to robotics, including manipulation [22], are also being actively explored.

In this work, the sensing principle, design, and variation of tactile image sensors employing cameras, with reference to studies that include recently published ones, are reviewed, and future developments are discussed. Tactile sensors employing cameras are often referred to as “vision-based tactile sensors” or “optical tactile sensors”. In this work, “tactile image sensor” is used to make it clear that the output of the sensor is obtained as an image sequence, including distributed tactile information through a camera.

## 2. Basic Structure of Tactile Image Sensor

**Figure 1:**
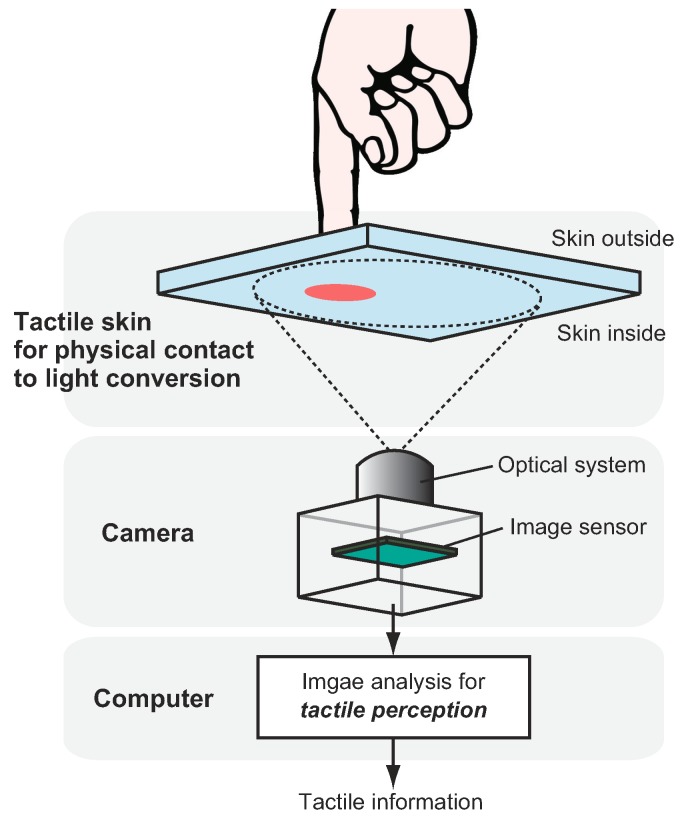
Typical structure of a tactile imaging sensor employing a camera. A tactile skin with a contact-light conversion mechanism somehow converts physical tactile stimuli into light signals, that can be captured by the camera. In a camera, an optical system that includes a lens determines spatial measurement range. The spatial resolution and the temporal resolution are determined by the specification of the image sensor. The image sequence, that is captured by the camera, is analysed by the computer on which the image processing software is running to extract tactile information.

Figure 1 shows the basic structure of a tactile image sensor employing a camera. The sensor consists of three major components: A tactile skin with physical contact-light conversion medium, camera, and computer. As the first step of the sensing, physical tactile stimuli should somehow be converted into a form of distributed light so that the camera can capture it as an image. There are several typical methods for that, as described in the next section. Spatial resolution, sensitivity, and measurable quantities often depend on the structure of this part. The camera, which is composed of an optical system and image sensor, observes changes on the surface backside of the tactile-light conversion medium. The measurement area and temporal resolution depend on the characteristics of the camera. Sometimes, an illumination system is required to make changes visible and/or clearer. After the image has been obtained, tactile information is extracted from the camera image through image processing, which is run on a computer. Which kinds of tactile information the sensor provides and the final performance of the sensor depends on the algorithm running on the computer and the performance of the computer. The major advantages of such a tactile image sensor employing a camera are as follows.

- High spatial resolution can be realized. The pixel number of the image sensor is now several to 10 mega pixels, even in an inexpensive one. Image sensors of 100 mega pixels are being applied to consumer digital cameras and smart phones. The pixel pitch on the image sensor is in the order of μm, and the resolution on the sensor surface can be controlled by an imaging lens. This feature leads not only to easily satisfy those in a human fingertip (mentioned in Section 1), but also to realize tactile function that is far beyond human’s tactile sensing ability, such as accurate measurement of the contact object shape.
- The measurement area can be controlled by an optical system. The view angle of the camera is determined by an imaging lens, and one can realize small and large measurement areas by choosing the appropriate lens. Smaller view angles provide higher spatial resolution. Additionally, one can use a special optical system developed in the field of computer vision to modify the measurement area. Details are provided in Section 4.
- The sensor surface is physically isolated from the camera. This feature leads to physical robustness and design flexibility for the sensor shape. Some examples are described in Section 4.
- Computer vision algorithms and tools can be used. Tactile information is extracted by analyzing the image provided by the camera. Here, one can use computer vision libraries, such as OpenCV [23], and machine learning (e.g., deep learning) frameworks to analyze the image and extract complex tactile information. Details are given in Section 5.

On the other hand, the most major disadvantage of the tactile image sensor is that a certain space is required between the tactile skin and camera, i.e., imaging distance, and the lens and image sensor, i.e., focal length. Therefore, the sensor tends to be thick. There are some efforts to thin the sensor to fit it to robotic fingers. Details are mentioned in Section 4. Another disadvantage is the high cost of image analysis for extracting tactile information from the image. However, recently, this cost is declining and the barrier to practical applications is disappearing.

## 3. Physical Contact to Light Conversion

**Figure 2:**
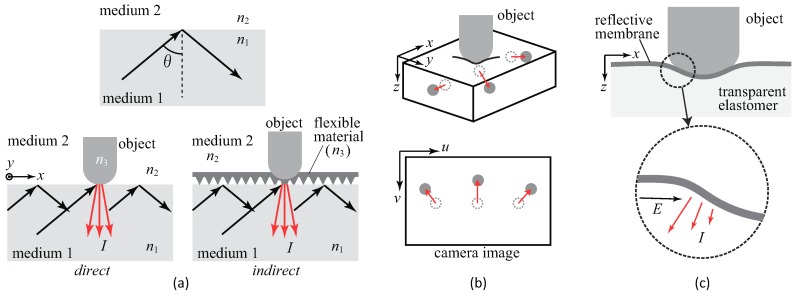
Principles of the three typical methods for converting physical tactile stimuli into light signals. (**a**) Light conductive plate-based method. The total reflection occurs if the incident angle θ is larger than the critical angle. When the object ($n_{3}$) makes contact with the sensor (medium 1), the condition for total reflection is broken at this part, and the scattered light (shown by the red arrows) is observed (direct method). In the case of using a flexible cover, this cover makes contact with the sensor (indirect method). (**b**) Marker displacement-based method. The markers (shown by the gray circles) move from the initial position (dotted circles) according to deformation of elastomer. These movements can be observed as two-dimensional motion on the camera image. (**c**) Reflective membrane-based method. The deformation of the sensor surface, which is coated by the reflective membrane, causes a gradient change at each point on the surface. The intensity of the reflective light changes depending on the gradient changes.

Converting the physical contact into light is the most important part of tactile sensing using a camera. The mechanism of this part determines how the light is provided to the camera. While there have been many studies on tactile sensors using cameras, and the conversion methods vary, most of them can be classified into the typical three categories according to the conversion method from the physical tactile stimuli to the light signal that can be captured by a camera. Table 1 summarizes these three categories, and Figure 2 shows more detailed principles for these conversion methods. Details are described in the following sections with reference to the table and figure.

### 3.1. Light Conductive Plate-Based Method

A light conductive plate can be used for tactile sensing employing a camera and was used in earlier studies [19,20,44,45]. The typical structure is shown at the top left in Table 1. Transparent acrylic board, glass, and silicone rubber can be used as a light conductive plate. Illuminated light from the light-emitting diodes (LEDs) is introduced to the transparent light conductive plate. For incident light with an incidence angle larger than the critical angle (defined by the refractive index of the medium), total internal reflection occurs, and the light is reflected inside the light conductive plate. When an object contacts with the surface of the light conductive plate, the light is reflected by the object at the contact point, which causes the light to scatter. This scattering is captured by the camera.

Figure 2a shows the principle of this method. When light enters from the medium with a larger refractive index (medium 1) to another medium with a smaller refractive index (medium 2) with the incident angle of θ, total reflection occurs if the incident angle is larger than the critical angle. Critical angle $\theta_{m}$ is represented as follows.

(1) $$\theta_{m}=\sin^{-1}\frac{n_{1}}{n_{2}}$$

where $n_{1}$ and $n_{2}$ are the refractive indices of media 1 and 2, respectively. So, the condition of total reflection is:

(2) $$\theta >\theta_{m}.$$

If the condition of the total reflection is not fulfilled, the light inside the light conductive plate goes out of the plate. When this condition is broken by the contact of the object, of which the refractive index is $n_{3}$ ($>n_{2}$), with the plate surface, the leaked light is applied to the surface of the object, and reflective light *I* occurs depending on the optical characteristics of the surface of the object.

(3) I(x,y,λ)=ρ(λ)E(x,y,λ)

where ρ(λ) is spectral reflectance with a wavelength λ, and *E* is the incident light to the object surface, which is leaked from the light conductive plate. The reflectance light is observed by the camera.

If one uses an acrylic plate as the light conductive plate, $n_{1}$ is approximately 1.5. For use in air, $n_{2}$ is 1.0. In this case, $\theta_{m}$ is approximately 42${}^{\circ }$. Generally, more light can be reflected if the critical angle is smaller. So, a higher refractive index of material of light conductive plate can be better for higher sensitivity. For underwater use, $n_{2}$ is 1.33 and $\theta_{m}$ is approximately 60${}^{\circ }$. A higher refractive index of the light conductive plate material is preferable in this case as well [39].

This method is used in [24,46,47,48,49]. Using infrared light as the illumination light makes it possible to separate the incident light to the camera into the visible light from outside the light conductive plate and the infrared light occurring by contact with the wavelength (λ in Equation (3)). The combined contact and proximity sensor in [25,50,51] uses this method for contact detection. Moreover, this method to detect contact using the light conductive plate has been used for multi-touch detection sensors in the field of human–computer interfaces [52,53].

**Figure 3:**
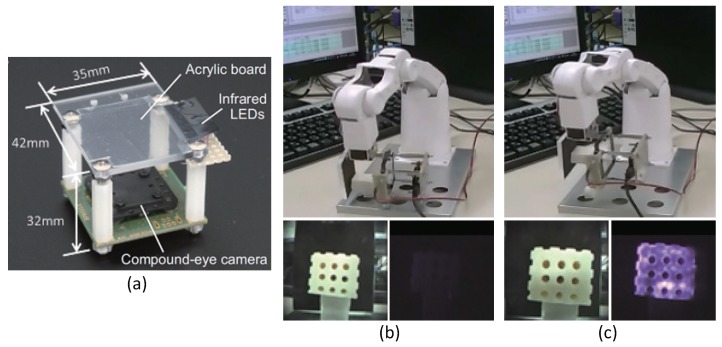
Example of tactile image sensor using a camera based on the light conducted plate. (**a**) Picture of the combined contact and proximity sensor (©IEEE 2016. Reprinted with permission from [25]); (**b**,**c**) application to robotic grasp control. The bottom row shows the output images including the contact detection (shown by purple color) and the visible light image of the object in the gripper. The contact detection is based on the direct light conducted plate method.

This structure can be divided into indirect and direct methods, as shown in Figure 2a. In the indirect method, a bumpy elastomer cover is put on the light conductive plate, as shown at top left in Table 1, and the object does not contact the plate directly. The advantage of this is that ρ and $n_{3}$ can be specified, because the contact object is known, that is, the elastomer cover. Based on this, force can be estimated [19] because, when an object is pressed against the plate with stronger force, the contact area is increased microscopically, and the brightness is increased. The disadvantage is that the measurement point is limited to the point of the bumps. The direct method does not use the elastomer cover, and the object directly contacts the light conducting plate. Figure 3 shows the example of a tactile image sensor with the direct light conductive plate-based method [25]. The contact area appears with single-pixel resolution, and the shape of the contact object can be seen clearly.

The light conductive plate method, especially the direct method, does not require the deformability of the sensor surface, in terms of the sensing principle, and a rigid material, such as aclyric plate, can be used as tactile skin, although two other methods, the marker displacement and the reflective membrane methods, require deformable tactile skin because these methods detect contact based on a geometrical change of tactile surface that occurs by contact. Generally, the deformability of the sensor surface can be important in robotic grasping [1]. However, this feature of the light conductive plate method is completely different from other methods, of which sensing is based on a geometrical change of the skin, and might be useful in specific environments such as underwater where ambient pressure applied to the sensor can be changed depending on the water depth [51].

### 3.2. Marker Displacement-Based Method

The marker displacement-based method is the most popular method to make tactile image sensors using camera. The typical structure of the sensor with this method is shown at the top middle in Table 1. Typically, a transparent elastic body in which several markers are arranged is put in front of the camera [26,29,30,31,34,54,55,56,57,58,59,60,61,62,63,64,65]. In [28,66], a fluid-type touch pad was proposed. The surface of the hemispherical touchpad is made of an elastic membrane, and the inside of the membrane is filled with translucent water. A dotted pattern is printed on the inside of the touchpad surface. Recently, it has become possible for soft materials to be processed by 3D printers, and the elastic part with markers can be directly fabricated through 3D printing [27,35,67,68,69,70,71,72].

**Figure 4:**
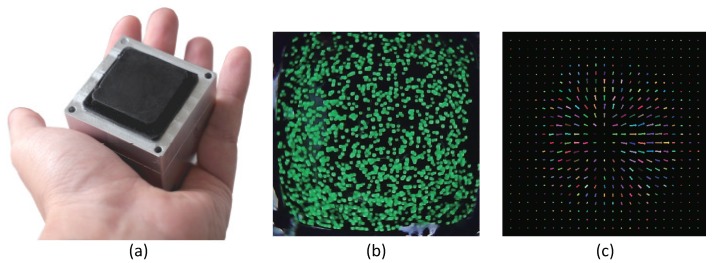
Example of tactile image sensor using a camera based on the marker displacement method. (**a**) Picture of a full-resolution optical tactile sensor developed by ETH Zurich research group [26]; (**b**,**c**) are the original image and dense optical flow computed with the DIS algorithm. Note that the flow is estimated at each pixel, and a subsampled version is shown in (**c**) for ease of visualization [26]. (Source: C. Sferrazza and R. D’Andrea, “Design, Motivation and Evaluation of a Full-Resolution Optical Tactile Sensor,” *Sensors*, 19, 2019 [26]).

Figure 2b shows the principle of this method. Markers embedded in the transparent deformable elastomer move when the object is pressed onto the surface of the elastomer and the elastomer deforms. Suppose the position of marker *i* at time *t* in physical coordinates is ${\left[x_{i}\left(t\right),y_{i}\left(t\right),z_{i}\left(t\right)\right]}^{T}$. In the image obtained from the camera, one can see the movement of each marker. The position of marker *i* at time *t* in the image coordinates is ${\left[u_{i}\left(t\right),v_{i}\left(t\right)\right]}^{T}$. For a monocular perspective camera, the relationship between the position of the marker in the physical coordinates and the image coordinates is:

(4) $$u_{i}\left(t\right)=\left[\begin{array}{c} su_{i}\left(t\right)\\ sv_{i}\left(t\right)\\ s \end{array}\right]=P\left[\begin{array}{c} x_{i}\left(t\right)\\ y_{i}\left(t\right)\\ z_{i}\left(t\right)\\ 1 \end{array}\right]$$

where *P* is the perspective camera matrix, and *s* is an arbitrary scale factor. In most cases, changes of the marker position from the initial state (with no contact) $u_{i}\left(t\right)-u_{i}\left(0\right)$ or changes between subsequent frames $u_{i}\left(t\right)-u_{i}\left(t-\Delta t\right)$ are computed to know the movement of the markers. By following the markers and measuring their displacement on the image from a camera, several kinds of tactile information, such as force, slip, and shape, can be obtained. Figure 4 shows an example of a tactile image sensor with the marker displacement method [26]. Each marker can be tracked on the image plane, and the optical flow reflecting the deformation of an elastic body can be computed.

In Equation (4), marker displacement in the *z* direction can not be measured directly. In [29,30,59], markers arranged in two different depths in transparent elastomer were used for estimating the deformation in the *z* direction. In [26], machine learning technology was used to estimate the marker position in *z* from two-dimensional optical flow input. In [37], the change in diameter of the markers was used to measure the movement in the *z* direction. In [73,74], a depth camera is used to measure the shape of the soft fingertip, including the *z* direction. The marker is not embedded in the soft fingertip body, but the depth camera projects patterns on the inside of the sensor skin, and the principle is basically the same in this category.

The advantage is that the structure is simple, and it is relatively easy to make. Furthermore, the displacement of the marker reflects the deformation of the elastomer body, and it is easy to understand the correspondence of the marker displacement to such physical phenomena as push, shear, and slippage. In [37,40,41], these sensors combine the marker displacement method with the reflective membrane-based method described in the next section, and the marker displacement is used for obtaining the information about the deformation of the sensor surface.

The disadvantage is generally that the measurement point is limited to the number of markers. However, in a recent study [26], motion information at each pixel on the image is computed based on the dense inverse search (DIS) optical flow algorithm (Figure 4c) [75]. This exploits the full resolution of the camera, and the spatial resolution of the tactile sensing does not depend on the number of markers.

### 3.3. Reflective Membrane-Based Method

**Figure 5:**
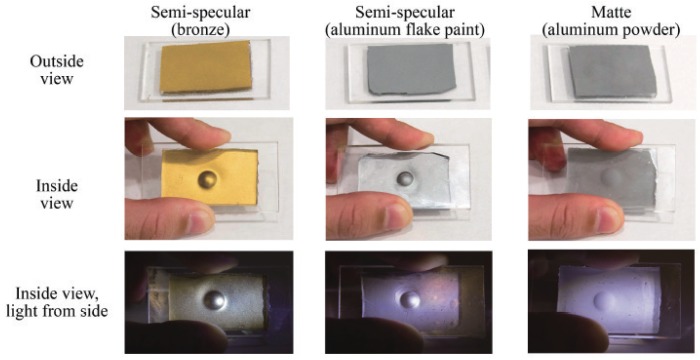
Comparison of appearance of tactile skin with three different reflective membranes used in GelSight tactile sensor developed by an MIT research group [40]. Three kinds of elastomer coatings: Semi-specular coating painted by bronze flake and aluminum flake paint, and matte coating by aluminum powder. In the second and third row, the three pieces of elastomer are pressed against a ball with diameter 6 mm, but the in the third row, the elastomer is illuminated by light from the side direction [40]. (Source: W. Yuan, S. Dong, and E. H. Adelson, “GelSight: High-Resolution Robot Tactile Sensors for Estimating Geometry and Force,” *Sensors*, 17, 2017 [40]).

The structure of a sensor with the reflective membrane-based method is shown in the top right of Table 1. The surface of the transparent elastomer sheet is coated with a reflective pigment, so that the illuminating light from the light source, such as LEDs, is well reflected. Figure 5 shows an example of the refractive membrane from [40]. When the object makes contact with the sensor surface, the elastomer surface deforms, and a shading image reflecting these deformations appears in the image obtained from the camera. This method has been used in GelSight, which is currently the most popular tactile image sensor [32,36,38,40,42,76,77,78,79,80], and some other sensors [37,41,43].

Figure 2c shows the principle of this method. By contacting the object, the sensor surface deforms. It is assumed that the sensor surface is very soft, and the shape of the object is completely reflected on the shape of the sensor surface. Here, we represent the surface using height function, z=f(x,y) [42]. By assuming a diffuse reflective surface, the intensity of reflection light *I* at (x,y) is expressed as:

(5) $$I\left(x,y\right)=R\left(\frac{\partial f}{\partial x},\frac{\partial f}{\partial y}\right)E\left(x,y\right)$$

where *R* is a reflective function that is defined based on the gradient of surface ∂f/∂x and ∂f/∂y, and *E* is incident light. For a Lambert surface, *I* depends only on the normal direction of the surface, if the direction of *E* is fixed. Practically, even if the sensor surface is not a complete Lambert surface, the intensity of the reflectance light can be changed by changing the gradient of the surface. Therefore, it is possible to obtain information about the deformation of the sensor skin, occurring by contact, as the local intensity change in the image.

The greatest advantages of this method are high sensitivity and resolution. By using a soft and thin reflective membrane, even a very small height difference, such as a very small raising of ink on a bill, can be detected in the image [76]. As shown in Equation (5), the information about the surface gradient is included in every pixel, and this sensor can measure not only the outline shape of the object [38] but also the surface texture [36]. Thus, this would be the most suitable method for measuring the precise position and orientation of the contact object. Moreover, using three images captured under three different light conditions enables the three-dimensional shape of the surface to be reconstructed based on photometric stereo. In [42], three different colors of illumination were applied to obtain three such images.

### 3.4. Other Methods

Some other methods that are not categorized as any of the three typical methods mentioned above and that use the camera and the nature of light have been also reported.

Total internal reflection is used to confine a light in the transparent plate in the light conductive plate method, as described in Section 3.1. In [81,82], the total internal reflection was used to project a pattern onto the surface inside the tactile skin, which is made of transparent elastomer. The pattern projected on the sensor surface provides image feature points, and it is captured by camera and used to reconstruct the shape of the sensor surface. Furthermore, they proposed to employ active patterns, which are presented by the LCD display and dynamically changed. The pattern was generated so that the sensor reconstructed the most precise surface deformation by using its reflection [82]. From the viewpoit of the sensing principle that the deformation of the elastomer body is measured as the displacement of an image feature, this would be similar to the marker displacement method. However, this work has the advantage of being able to change the pattern dynamically. In [83], a similar sensor structure was used, although diffusive light is applied to the surface inside the skin, instead of the pattern, and intensity distribution of reflected light was observed by camera. Moreover, the specular reflective surface has been often used to measure very small deformations in minute domains [84,85]. In [85], a plate with a special printed pattern consisting of crossed line gratings was prepared, and the pattern was reflected by the specular surface and imaged by the camera. The mechanical deformation causes a slight distortion of the pattern image. Through image analysis, a small deformation less than μm was detected. In [84], a cantilever displacement was measured by detecting the deflection of a weak laser beam which is reflected by the mirror attached to the lever. The deflection was sensed with an apposition-sensitive detector (PSD). This was used for sample measurements under the microscope. These works are for shape measurement, not tactile sensing; however, they is very interesting and closely related to the tactile image sensors because they can optically measure minute displacements of the surface.

Photoelasticity is the interesting optical phenomenon that the optical properties of a material change under small mechanical deformations, and it has been used to visualize and analyze the stress distribution in material and structures. In [86], a tactile sensor based on the photoelastic effect, which is capable of detecting object slip as well as providing normal force information, was proposed. Light emitted by a source is passed through the photoelastic layer located between a polarizer and an analyzer. On entering the stressed transparent photoelastic material, the polarized light is split into two components, which vibrate in the two perpendicular planes of principal stresses. The two rays emerging from the material are received by an analyzer, which only transmits the components of two rays in its plane of polarization. This causes a change in the light intensity at the receiver [86]. This study used a photodiode as a receiver; however, a camera as well can be used for the receiver from the viewpoint of the principle.

In [21], a bundle of optical fibers was put under rubber skin with a reflective surface. The reflective light intensity changed depending on the deformation of the rubber skin, and it was transmitted to the camera through the optical fibers. In [87,88,89], a sensing structure that consists of an elastic body, a connector with a mushroom shape, and rubber membranes is proposed. When external force is applied to the elastic body, the force is transmitted to the rubber membrane through the connector, and the membrane deforms. Illuminating the membrane with an LED from the side enables the reflected light image reflecting the film deformation to be obtained for use in estimating the force vector. In [90], a fiberoptic sensor that can be used in a magnetic resonance imaging (MRI) environment is proposed. For that, no metallic materials are used. An arrayed sensor with 3 × 3 elements was fabricated, and each element consisted of a mirror and two optical fibers—one for transmitting the light and the other for receiving the reflected light from the mirror. When a force is applied on the sensing element, the displacement change between fiber and mirror causes a change of light intensity in the receiving fiber. In [91], the sensor consisted of a transparent elastomer surrounded by eight translucent elastomer parts with conical feet. The surface pattern of the contact object is seen through the transparent elastomer, and force is evaluated based on the shape and displacement of the image of the eight conical feet, captured through a light conductive plate. In [92,93], multiple color filters and multiple LEDs with different colors were used, respectively, to obtain the deformation of sensor skin as color changes. In [92], a subtractive color mixing process using yellow and magenta translucent markers placed at different depths in the transparent elastomer body was used to estimate the three-dimensional displacement field. If tactile information, such as force, is encoded as intensity or color information in each pixel value, the computational cost can be low because we can just see each pixel value to know the tactile information. The tactile sensing in [94] was composed of a camera and an insensitive flexible beam of which the force-deformation characteristic is known. When the beam makes contact with the object, the camera observes the deformation of the beam that includes the information about force applied to the beam and the distance from the object. The sensor with whiskers in [95] detects the contact with the whiskers using a camera based on the marker displacement-based method.

## 4. Shape of Tactile Skin and Sensor Size

**Figure 6:**
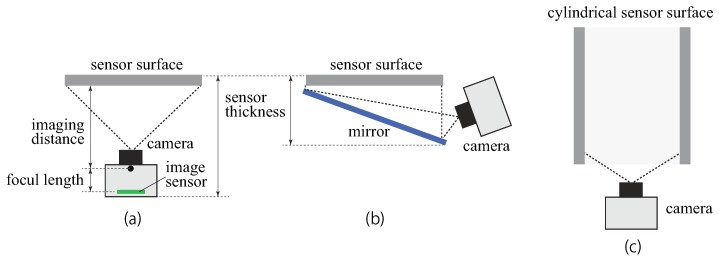
(**a**) Standard shape of the sensor with the flat sensing skin. (**b**) Reducing the thickness of the sensor using a mirror in [101]. (**c**) The inside of the cylinder can be seen using a perspective camera, such as that used in [97,98].

The most common shape of the sensing skin in the tactile image sensors is one that uses a sensor surface with a flat plane (Figure 6a). The camera is put under the tactile skin and observes the backside of the skin surface. For the marker-based method, it is easy to make the sensor skin surface a curved one that is suitable for robotic fingers [30,67]. Moreover, for the light conductive plate method, it is possible to make a curved sensor surface [45], although the condition for the total reflection (Equation (2)) has to be met. For the reflective membrane method, it is difficult to make the sensor surface curved due to its optical principle.

One of the advantages of using a camera for tactile sensing is that its measurement area can be varied, because it is sufficient if there is a measurement area within the field of view of the camera. The field of view of the camera can be controlled by devising an optical system, including an optical lens and mirror. For deploying to larger planes and narrower planes (for higher spatial resolution), we can choose the optical lens with the appropriate view angle and the imaging distance, although there might be some difficulties to fabricate a large tactile skin for the light conductive plate method and the reflective membrane method. A sensor with a cylindrical shape is an interesting example of that (Figure 6c) [96,97,98]. By observing the inside a sensor body that has a cylindrical shape with a camera with wide viewing angle, such as a fish-eye lens, tactile information about the entire surface of a cylindrical pipe can be detected. A cylindrical configuration is useful for a tactile sensor for a link of a robotic manipulator [98], applications for capsule endoscopy [97], and determining curvature [99]. An omnidirectional camera [100] also can be used in this configuration.

**Figure 7:**
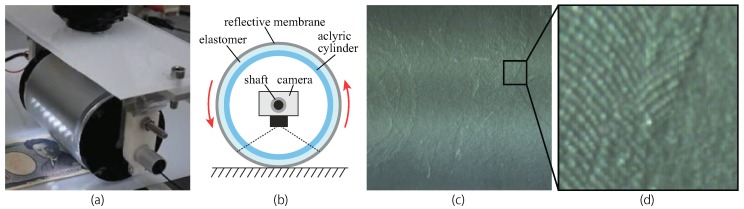
Rolling cylindrical tactile image sensor. (**a**) Picture of the sensor rolling on the surface of the object (a Japanese bill), (**b**) structure of the sensor, (**c**) output image obtained through a image mosicking, (**d**) enlarged output image. The video of the experiment is available: https://youtu.be/6mm4fgTJWB0.

Another advantage for devising the configuration of the sensor is that the sensor surface and camera are physically separated. This means that the sensor surface can move relative to the camera. A rolling tactile image sensor is an example of that (Figure 7). The camera is put inside the cylindrical pipe, which forms a reflective membrane-based sensor surface. By rolling this sensor body on the target plane, the texture of the surface is smoothly and continuously obtained, even for a large observation area. Through image mosaicking, the whole tactile image of the target plane can be reconstructed with high spatial resolution.

The major constraint in a tactile sensor using a camera is that a certain space is necessary between the sensor surface and camera lens (imaging distance) and the lens and image sensor (focal length) (see Figure 6a). This causes the sensor to become thick. Robotic grippers often require a thin tactile sensor so that the manipulation is not disturbed by the thickness of the gripper or finger. In [101,102], a thin sensor configuration was reported. A mirror was used to reflect the sensor surface to the camera placed beside the sensor surface (Figure 6b). The thickness of the finger equipped with the sensor was 20 mm, and it had a slim fingertip, so it could be inserted between the target object and non-target object in a cluttered environment.

In [25,51], a compact and thin multiple camera system, called a “compound-eye camera”, [103,104] was used (see Figure 3a). This consisted of a lens array, a signal separator, and an image sensor. It was constructed as an array of elemental parts called “units” that included a lens and a small segment of the image sensor. The compound-eye camera used in [25] had an array of nine units. Such a development of the optical system is also useful for reducing the size and thickness of tactile image sensors.

## 5. Image Analysis for Extracting Tactile Information

Tactile information is extracted from the camera image through image analysis. Since how the tactile stimuli are reflected in the camera image depends on the principle of the physical contact-to-light conversion method, there are typical image analysis algorithms for each method.

For the light conductive plate method, the contact area is represented by a brighter pixel value, so it can be extracted by thresholding ([25], for example). For the extracted area, one can compute the area, number of blobs, shape, and so on. In the indirect method, the material of the contact object is known—that is, an elastomer cover. In this case, it is possible to estimate the applied force from the pixel intensity in the image by calibrating the relationship between the load and brightness in advance [19].

For the marker displacement method, the typical method is to extract the optical flow by tracking each marker on the image plane. Moreover, in [27], Voronoi tessellation was used for better visualization of the displacement of many markers and for analyzing them. These image features are used in post-processing to extract various pieces of tactile information, such as three-dimensional deformation of the elastomer, force applied to the elastomer part, and so on. In [29,59], a three-dimensional forcefield was estimated based on elasticity theory. To compute the three-dimensional force vector from the two-dimensional marker displacement on the image plane, two layers of markers, which have different colors for different layers, at separate depths are used. In [102], normal force distribution was estimated based on the inverse finite-element method (FEM), which is effective at reconstructing the external force applied. This sensor is categorized into the reflective membrane method; however, the force estimation is based on marker displacement. Measurements of shear and slip in [32] are also based on the marker displacement method.

For the reflective membrane method, the camera image includes a fine surface texture and clear outline shape of the contact object. In [37,38,80], in-hand object localization was performed through pattern matching. A typical reflective membrane-based sensor, GelSight, was applied for many tactile sensing tasks, such as recognizing surface textures [36].

Artificial deep neural network is a powerful tool for extracting complex tactile information from an image. In [26], an artificial deep neural network was used to perform the tactile sensing task, such as estimation of the normal force distribution applied to a soft material. In [77], information on hardness was estimated from the change of the object shapes and contact force through recurrent convolutional networks.

## 6. Combined Sensing of Multiple Modalities

**Figure 8:**
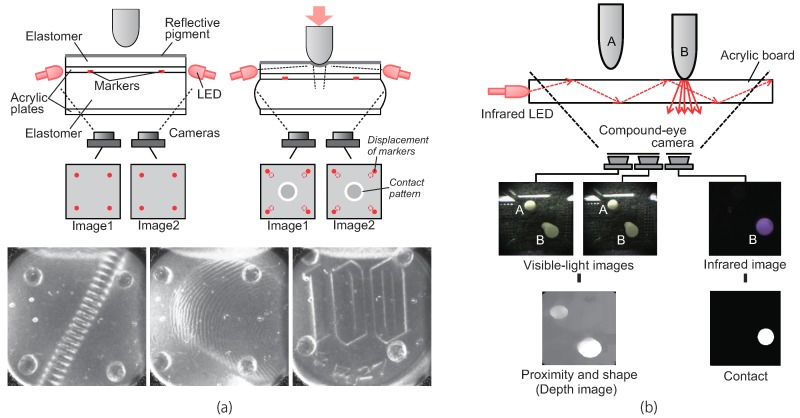
Examples of combined tactile image sensor. (**a**) Structure of the sensor for combined force and surface texture sensing. Force sensing and surface texture imaging is based on the marker displacement and the reflective membrane method, respectively. The bottom three images show texture images, a screw, a fingertip, and a coin (©IEEE 2018. Reprinted with permission from [37]). (**b**) Combined contact and proximity sensing. Proximity and the three-dimensional shape of the object are detected from visible-light stereo pair images. Contact and its area are detected from infrared image (©IEEE 2016. Reprinted with permission from [25]). A compound-eye camera, compact, and thin multiple camera system, is used to obtain both visible and infrared light images through the different optical filters.

Each of the typical three methods for converting physical contact into light information mentioned above has different features because of different transduction principles. By combining some of these methods, the benefits of those multiple methods can be used. The sensors in [32,37,41] combined the marker displacement method with the reflective membrane-based method to obtain both a tactile texture image and marker displacement reflecting the deformation of a flexible sensor body. Figure 8a shows the example of such a combined tactile sensor in [37]. The image obtained based on the reflective membrane method is used for measuring the position and orientation of the object, and marker displacement is used for estimating the external normal and shear force. By using a six degrees-of-freedom robotic arm and a robotic hand equipped with the proposed sensor, the robot motion for inserting a screw into a screw hole and temporary tightening can be achieved based on only tactile information obtained from the proposed sensor device.

The sensors focused on in this work are used to obtain tactile information through physical contact with the target object. However, in the robotic grasping process, it is often necessary to use different types of sensors to obtain information about the object to be manipulated. In fact, a robotic hand equipped with tactile and proximity sensors has been developed for grasping a large variety of objects [105,106]. In [25], a combined proximity and tactile image sensor was proposed. Tactile information is obtained based on the light conductive plate method with infrared illumination light, and proximity is detected based on stereo matching with a pair of visible light images obtained through a transparent sensor surface (Figure 8b). The robot motion for searching, approaching, and grasping can be controlled based on sensor information obtained only from the proposed combined device. Moreover, in [31,60], information about the target object can be obtained through a transparent sensor surface before making contact. Even if the force is extremely small and difficult to detect based on the deformation of the elastomer material, contact is detected based on depth measurement between the object and sensor surface.

In human haptic perception, both tactile as well as thermal information can play an important role. In a robotic system, thermal information is required for distinguishing two objects based on temperature and sensors in telepresence systems, for instance. In [107,108,109], sensing of the applied forces and temperature changes on the sensor surface were obtained simultaneously. For thermal sensing, the fingertip is covered by a thermo-sensitive material of which the color changes depending on the temperature of the contact surface. This information is obtained as an image, so one can know the position where the temperature is different from that of the surroundings. Force sensing is based on the marker displacement method.

## 7. Conclusions

The principle and typical designs of tactile image sensors employing cameras were described, and recent developments were reviewed. By using a camera, image-reflecting tactile information can be obtained with high spatial resolution. Tactile information is obtained through post-image analysis. One can employ computer vision technology, such as an open-source library and recent sophisticated algorithms, for extracting various types of tactile information. In particular, recent machine-learning technology, such as deep learning, is suitable for realizing complicated tactile perception through the image.

The most important component of a tactile image sensor is the sensor surface that converts physical contact into light signals. Flexible materials are often used for that because contact is detected based on geometrical change of tactile surface (except to the direct light conductive plate method). The deformability of the sensor surface can be generally important in robotic grasping. On the other hand, the mechanical and optical characteristics of the material affect the performance of tactile sensing, such as force measurement range and sensitivity, and this can be chosen depending on the task. In [33], two widely used typical tactile image sensors, FingerVision and GelSight, were compared from the view point of shear force sensing, and a calibration method was proposed to handle the range of forces needed for a specific manipulating task.

Cameras and image sensors are also important components. The specification of the camera is directly related to the performance of the tactile sensing. For example, the temporal resolution of tactile sensing is determined by the frame rate of the camera and the image analysis speed. The camera is the front end of the sensing, and so it gives the first bottleneck. A standard digital camera has several tens of frames per second (fps) in the frame rate, and this is relatively low compared with other conventional tactile sensors, such as strain gauge-based sensors. In general, sensor elements should respond as quickly as 1 ms for real-time tactile feedback robotic control [1], and this requires 1000 fps for the camera. Nowadays, high-speed cameras with up to 200 fps are available; however, faster cameras have a larger enclosure and are not suitable for embedded use. An event-based camera [110], which provides asynchronous event output, is an interesting imaging device for realizing higher temporal resolution with a small camera and low power consumption. In [111,112,113], this event-based camera was used for tactile image sensors. The temporal resolution in [111] was 500 μs.

In the field of robotics, especially for robotic perception and manipulation, integration of tactile and visual sensing is one of the interesting directions for utilizing tactile image sensors. Tactile image sensors provide high-density distributed tactile information in the form of an image, which is suitable for combining with vision sensor output, which is a form of image as well. In [47,114,115,116,117], tactile and vision sensing were combined for object tracking, cloth texture recognition, three-dimensional shape perception, and estimation of grasp success probability.

The tactile image sensor employing a camera is expected to develop further along with the development of related technologies, such as image sensors, optical devices, and image processing hardware and software, and to be used for a wide variety of applications, such as robotic sensors, computer interfaces, inspection, and medical measurement.

## Figures and Tables

**Table 1:** Typical structures and features in typical three categories of tactile image sensors employing a camera, in terms of the conversion method from physical contact to light signal.

Method	Light Conductive Plate	Marker Displacement	Reflective Membrane
**Structure**	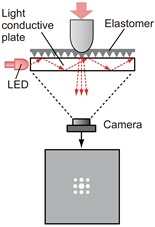	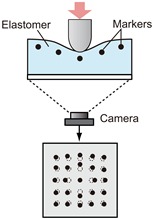	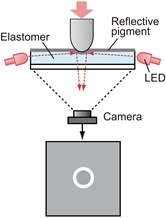
**Physical quantity to be changed by physical contact**	Refractive index of the material that makes contact with the light conductive plate (Equations (1)–(3))	Marker position (reflecting a geometrical change of flexible material) (Equation (4))	Surface gradient (reflecting geometrical change of flexible material) (Equation (5))
**Typical measurable quantities**	Contact position and area [19,24,25]/force ** [19,24]	Contact position [26,27,28]/force [29,30,31]/shear [27,29,30,31,32,33]/torque [31]/slip [32,34,35]	Contour and surface texture of contact object [36]/position and orientation [37,38]
**Typical post image processing**	Thresholding [19,39]	Tracking [29,31]/optical flow computation [26]	Pattern matching [37,38]
**Advantages**	High spatial resolution (at the imager’s resolution) */ease to detect contact (just seeing pixel value change)	Easy to make/no special lighting arrangement is required/arbitrary shape of sensor skin is possible	High spatial resolution/can obtain fine surface texture (such as fingerprint)
**Disadvantages**	The response depends on optical characteristics of the object */less spatial resolution in the case of use of the elastomer cover **	Measurement points are determined by markers (but can be improved via post processing [26])	Does not respond to the object without any edge or texture (such as flat object which is larger than sensing area)
**Remarks**	Does not require deformability of the sensor skin in direct method *, but some indirect methods use deformability of elastomer cover ** [19]	Can be combined with the reflective membrane method [37,40,41]	Three-dimensional shape reconstruction is possible based on photometric stereo [42] or with sensor movement [43]
